# The protective effects of angelica organic acid against ox-LDL-induced autophagy dysfunction of HUVECs

**DOI:** 10.1186/s12906-020-02968-7

**Published:** 2020-06-01

**Authors:** Xuefeng Li, Jing Zhou, Yinghuan Dou, Yanbin Shi, Ying Wang, Jianli Hong, Junnan Zhao, Jiaying Zhang, Yang Yuan, Mengru Zhou, Xiangxiang Wei

**Affiliations:** 1 School of Basic Medical Sciences, Lanzhou University, Lanzhou, 730000 China; 2 Gansu Provincial Hospital, Lanzhou, 730000 China

**Keywords:** Angelica organic acid, Ferulic acid, Ox-LDL, Autophagic flux, Atherosclerosis

## Abstract

**Background:**

Angelica root is the dry root of the Umbelliferae plant *Angelica sinensis (oliv) Diels*. Angelica organic acid (OA) is the main active ingredient in Angelica sinensis, and it exerts potential anti-atherosclerotic effects by preventing Oxidized low-density lipoprotein (Ox-LDL) induced endothelial injury. To study the protective effects of OA on ox-LDL-induced HUVECs autophagic flux dysfunction and inflammatory injury.

**Methods:**

OA were isolated by water extraction and alcohol precipitation, and then the content of ferulic acid (FA) in the OA was determined by high performance liquid chromatography. The ox-LDL-induced endothelial injury model was established. The effect of ferulic acid on the survival of Human umbilical vein endothelial cells (HVUECs) was detected by CCK-8 assay. HUVECs were pretreated with different concentrations of OA (20 μmol/L, 40 μmol/L, and 80 μmol/L), and Western Blot was used to detect the expressions of LC3II, p62, MCP-1, VCAM-1 and LOX-1. The autophagosomes in HUVECs were observed by transmission electron microscopy (TEM).

**Results:**

20 μmol/L OA could increase the expression of LC3II and decrease the expression of p62, MCP-1, VCAM-1 and LOX-1. The results of TEM showed that angelica organic acids promoted cell organelle degradation in autolysosomes.

**Conclusion:**

OA could reduce inflammation, protect endothelial cells and play an anti-atherosclerotic role by enhancing the autophagy flux of damaged endothelial cells, in which FA the major active ingredient of OA played a major role.

## Background

Ox-LDL can lead to the formation and progression of atherosclerotic plaque by inducing activation and dysfunction of endothelial cell, formation of macrophage foam cell, and migration and proliferation of smooth muscle cell [1]. A large number of ox-LDL accumulations are found in advanced atherosclerotic plaques and ox-LDL-induced autophagy dysfunction is observed [2]. Autophagy in cell includes the degradation of dysfunctional organelles and long-lived proteins [3]. In endothelial cells, moderate autophagy can reduce inflammation and oxidative stress, and promote the removal of damaged organelles, such as depolarizing mitochondria [4]. In Wip1-deficient mice, the excision of the Wip1 phosphatase lead to the activation of autophagy, inhibition of macrophage conversion into foam cells and prevention of atherosclerotic plaque formation [5]. Therefore, induction of autophagy may be an important determinant of atherosclerotic plaque stability [6].

OA has obvious protective effect on endothelium and anti-atherosclerotic. Its main mechanism is to inhibit the expression of endothelial adhesion molecules and reduce oxidative stress and inflammation [7, 8]. There have been no reports on the effect of OA in regulating autophagic flux against atherosclerosis.

This study was done to investigate the effect of ox-LDL on autophagic flux of HUVECs and look into whether OA could exert anti-atherosclerotic by enhancing the injured autophagic flux in HUVECs.

## Methods

### Preparation of OA extract

Angelica was bought from Min County, Gansu Province on August 2015. It was identified as the roots of Angelica sinensis (Oliv.) Diels by Dr. Jianyin Li from Lanzhou University School of Pharmacy. (The voucher specimen number of the stored herbarium specimen is 621125720823259LY.) The protocol for extraction of OA was as followings: (1) Angelica (100 g) was crushed and extracted with distilled water (800 ml) for 8 h. (2) The supernatant was collected and filtered through filter paper to obtain about 200 ml solution. (3) 500 ml absolute ethanol was added overnight and the precipitate (angelica polysaccharide) was removed. (4) The supernatant was collected, filtered, and the filtrate was concentrated by rotary evaporation in vacuum at 4 °C for removing ethanol. The residue was lyophilized. (5) The OA extract (23.35 g) was finally obtained and put in a closed glass vial at 4 °C for further use [9].

### High performance liquid chromatography analysis of OA extract

The major components of OA extract (ferulic acid) was identified and quantified through high-performance liquid chromatography (HPLC). C18 column (4.6 mm × 200 mm, 5 μm) (Diamonsil, China) was used as solid phase and the column temperature was maintained at 35 °C. At the same time, the mobile phase involves acetonitrile and 0.1% phosphoric acid solution (17∶83). while the monitoring wave length is 316 nm with a flow rate of 1.0 ml min^− 1^.

### Cell isolation and identification

Umbilical cords were obtained from the Department of obstetrics, Gansu provincial people’s hospital. The puerperae included were free from gestational diabetes, HIV and hepatitis, etc. Informed consent was gotten prior to collection. The approval of the agreement was awarded by the Gansu provincial people’s hospital ethics Committee and the Lanzhou University School of Basic Medicine ethics Committee. HVUECs were seperated by trypsinization [10]. Cells were spread out in 25 cm^2^ flasks (Corning, USA) and cultured in Dulbecco’s Modified Eagle Medium: Nutrient Mixture F-12 (DME/F-12; HyClone, USA) supplemented with 10% fetal bovine serum (Biological Industries, Israel), 100 U/ml penicillin and 100 g/ml streptomycin (HyClone, USA) and Endothelial Cell Growth Supplement (ECGS; ScienCell, USA). The flasks were incubated at 37 °C in a humidified atmosphere containing 5% CO_2_. Immunofluorescence technique was applied to detect the expression of von Willebrand factor (vWF). HUVECs were fixed with 4% paraformaldehyde for 10 min at room temperature. After washing with PBS (HyClone, USA) for 5 min, the fixed HUVECs were incubated for 1 h with 1% BSA (Solarbio, China) blocking solution. Then a primary antibody vWF (Boster, China) was used at a dilution of 1:50 for the samples, which were incubated overnight at 4 °C. To continue washing with PBS for 5 min, the samples were incubated with FITC-goat anti-rabbit IgG (Boster, China) at a dilution of 1:200 in blocking solution at room temperature for 1 h. With the last washing with PBS, the samples were mounted with DAPI and observed under the fluorescent microscope (OLYMPUS/DP73, Japan).

### Cell viability measurement

CCK-8(DOJINDO, Japan) was used to test cell viability according to the manufacturer’s operationguide. Shortly, HUVECs were seeded in a 96-well microplate at an proper density of cells/well, and then preprocessed with FA at a series of concentrations (0, 10, 20, 40,80and 160 μmol/L). Then, CCK-8 solution (10 μL/well) was put to the wells, the plate was incubated at 37 °C for 1.5 h, and the absorbance was examined with a microplate reader (BIO-TEK, USA) at a wave length of 490 nm. The optical density value was recorded as the percentage of cell viability in relative with the control group.

### Western blot analysis

Cell lysates were prepared using lysis buffer (Solarbio, China) with phenylmethylsulfonyl fluoride (PMSF; Solarbio, China). 10% sodium dodecyl sulfate-polyacrylamide gels were used to split the sample proteins and transferred on polyvinylidene fluoride membranes (Millipore, USA). The membranes were blocked with 5% (w/v) skimmed milk solution for 1 h, and incubated with rabbit primary antibodies (Abcam) against LC3II(1:2000), p62(1:50000), LOX-1(1 μg/mL), VCAM-1(1:10000), MCP-1(1:5000) and mouse primary antibodies against β-actin (1:2000) at 4 °C for 12 h. After that, TBST was used to wash the membranes three times, 15 min a time and then incubated with secondary antibodies for 1 h. Immune complexes were detected by MiniChemi (Beijing Sage Creation Science, China).

### Transmission electron microscopy

Cells were put in 6-well plates at 2 × 10^4^ cells/well. The cells were pretreated with OA for 2 h and then treated with 40 mg/L ox-LDL for 24 h. Then the cells were gathered and fixed with 2.5% glutaraldehyde. The samples were fixed in 1% osmic acid for 1 h, dehydrated by graded ethanol solutions. Then the cells were sent to the electron microscope room of Lanzhou University Medical Experimental Center for soaking, curing, slicing and radiography.

### Statistical analysis

Results were presented as means ± standard deviation (SD). The differences between groups were evaluated by one-way ANOVA and Bonferroni test. A *P* value of less than 0.05 was considered significant.

## Results

### Analyzing the chromatograms of FA in angelica organic acids

**Fig. 1:**
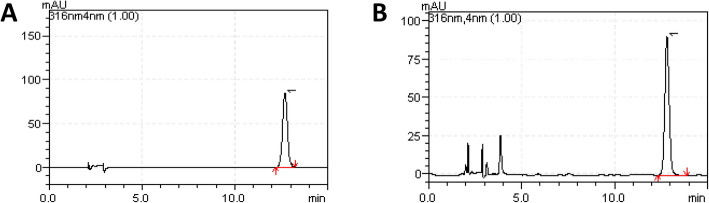
HPLC (**a**) FA standard (1), (**b**) OA sample including FA (1)

The test solution of FA and the standard solution had a peak at the same retention time (12.54 min). The number of theoretical plates > 5000 was calculated with FA chromatographic peak (Fig. 1) The obtained Angelica extract yield was 11.675 mg/g, the FA content was 1.392 mg/g, and the RSD was 0.318.

### Observation of the morphology and structure of HUVECs

**Fig. 2:**
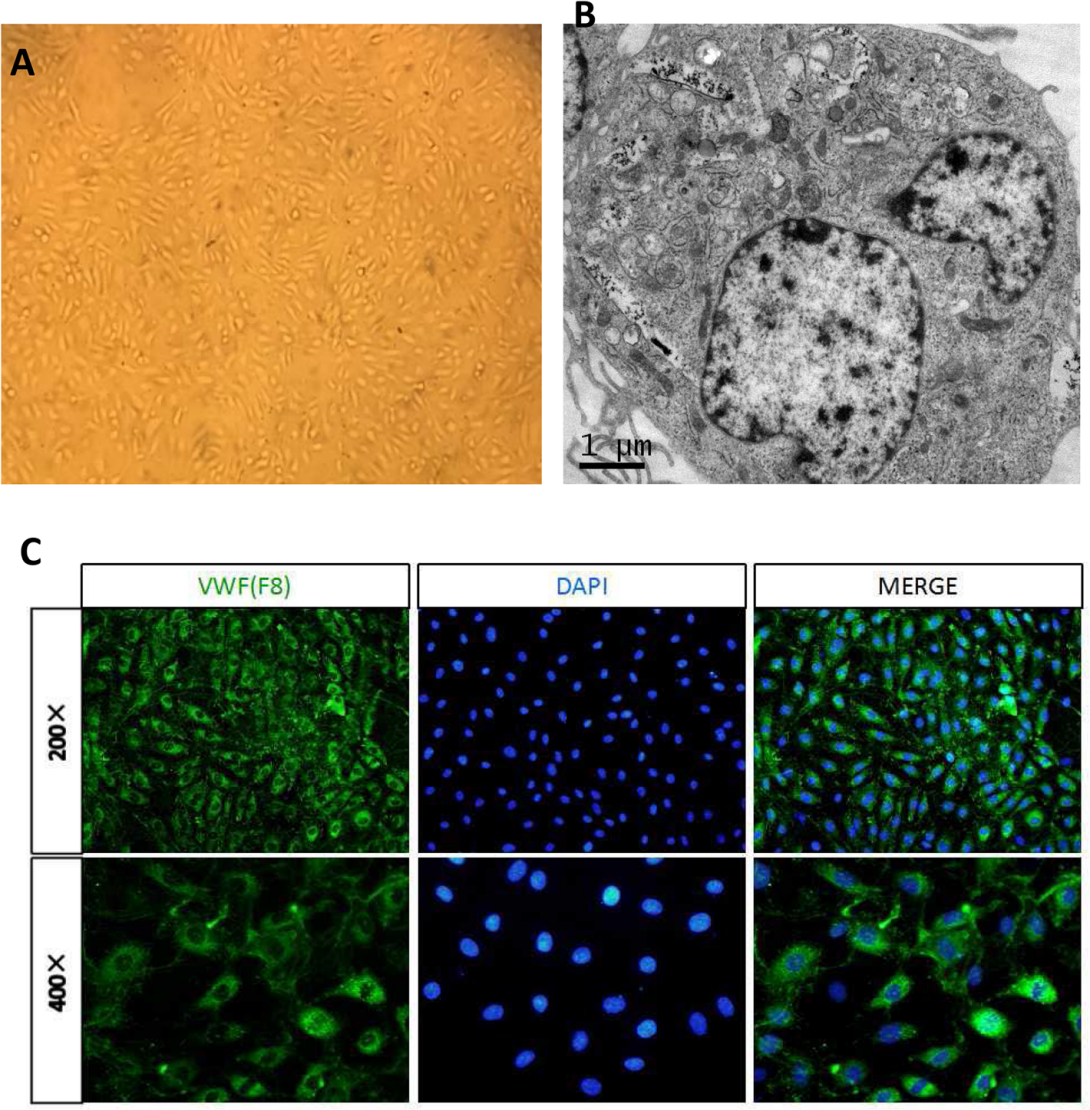
Observation of the morphology and structure of HUVECs. **a** Morphology of HUVECs. Cultured cells seperated from an umbilical cord looked like cobblestone by phase contrast microscope. **b** Ultrastructure of HUVECs. Cultured cells showed Weibel-Palade body by TEM. **c** Identification of HUVECs. The isolated cells were Factor VIII positive in immunofluorescence staining. The cultured cells were endothelial cells

Under the inverted microscope, most of the 2–5 generation HUVECs were round and elliptical, and the cells were arranged in a single layer like paving stones (Fig. 2a). Under the fluorescence microscope, VWF (F8)-related antigen immunofluorescence staining of HUVECs showed that the cytoplasm was rich, showing strong green fluorescence which is a positive reaction, indicating that the cells are endothelial cells (Fig. 2c). The Webel-Palade body can be seen in the cytoplasm of HUVECs (Fig. 2b) by TEM that are characteristic markers of human endothelial cells, which longitudinal section is long rod-shaped.

### Ox-LDL induces autophagic flux damage in HUVECs

**Fig. 3:**
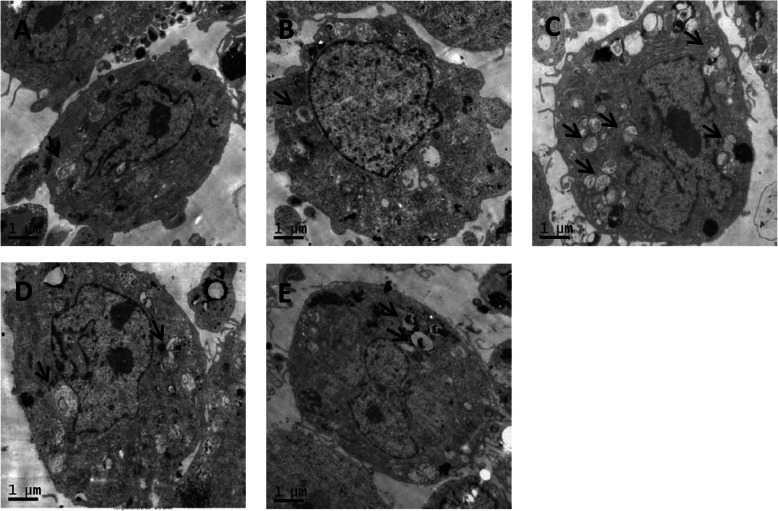
**a** is ox-LDL 0 mg/L group, **b** is ox-LDL 20 mg/L group, **c** is ox-LDL 40 mg/L group, and **d** is ox-LDL 60 mg/L group; the last group was ox-LDL 80 mg/L; the black arrow in the figure shows the autophagosome

The results of TEM showed that when HUVECs were treated with 20 mg/L ox-LDL, a small amount of autophagosomes appeared in the cytoplasm. When HUVECs were treated with 40 mg/L ox-LDL, the autophagosomes was the most in the cytoplasm. (Fig. 3) Therefore, in this experiment, the concentration of ox-LDL was selected to be 40 mg/L.

**Fig. 4:**
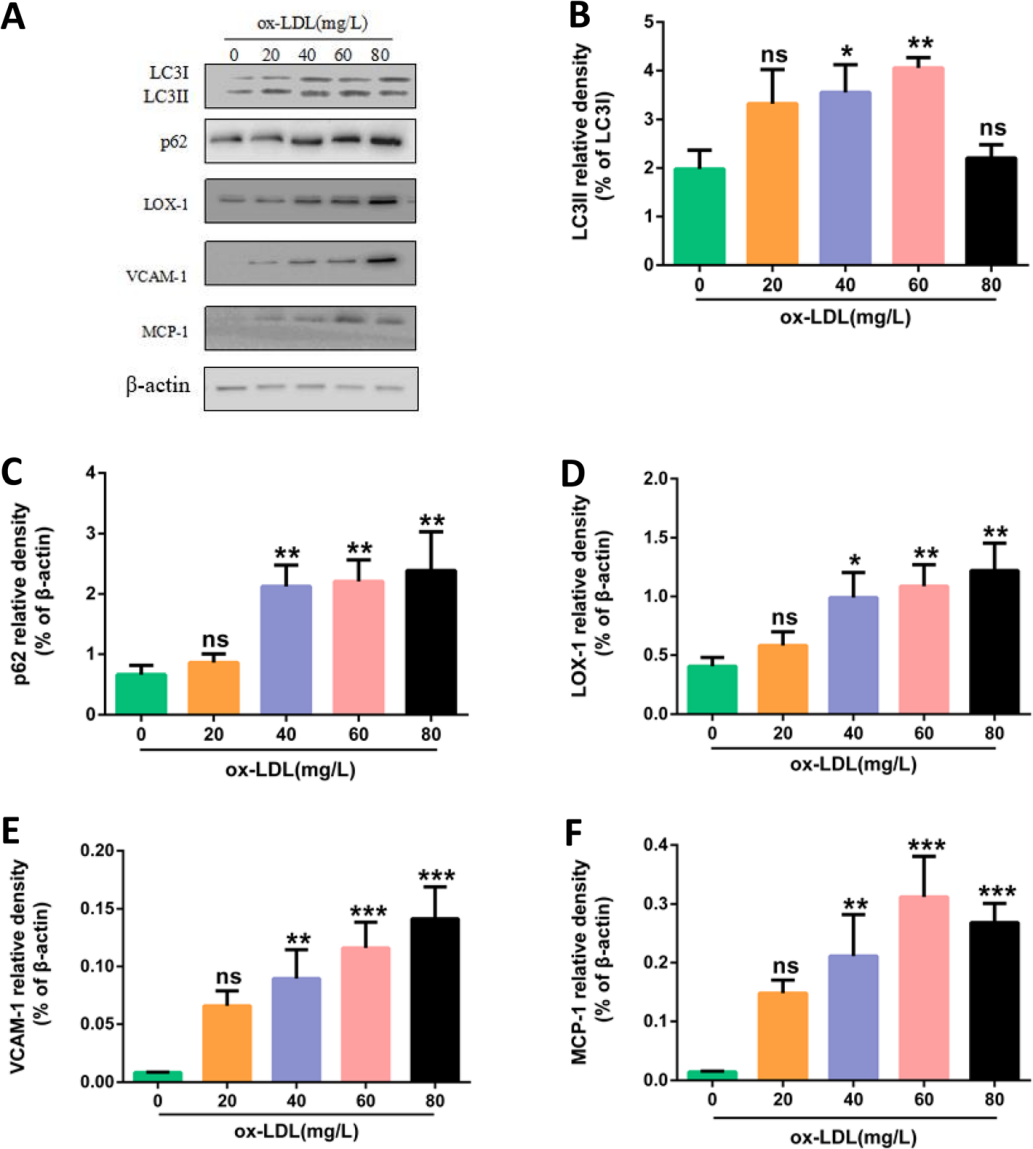
Different concentrations of ox-LDL induced the increase of inflammation and autophagy protein expression in HUVECs**. a** Protein expressions of LC3II, p62, LOX-1, VCAM1, MCP-1, β-actin in HUVECs. The cells were treated with 0 mg/L、20 mg/L、40 mg/L、60 mg/L、80 mg/L ox-LDL respectively for 24 h. **b**, **c**, **d**, **e**, **f** Bar charts show the mean intensity of every protein quantified and normalized versus β-actin expression. Values are submitted as mean ± S.D. based on seperate experiments in triplicate.(*) *P* < 0.05, (**) *P* < 0.01 and (***) *P* < 0.001 versus 0 h

HUVECs were treated with medium containing different concentrations of ox-LDL (0 mg/L, 20 mg/L, 40 mg/L, 60 mg/L, 80 mg/L) for 24 h. The results of Western blot showed: 40 mg/L and 60 mg/L ox-LDL treatment for 24 h increased the expression of LC3II protein (autotrophic marker protein) of HUVECs (*P* < 0.05 and *P* < 0.01); 40 mg/L, 60 mg/L, 80 mg/L ox-LDL treatment for 24 h increased p62 protein (autophagy-degraded substrate) expression of HUVECs (*P* < 0.01) (Fig. 4).

**Fig. 5:**
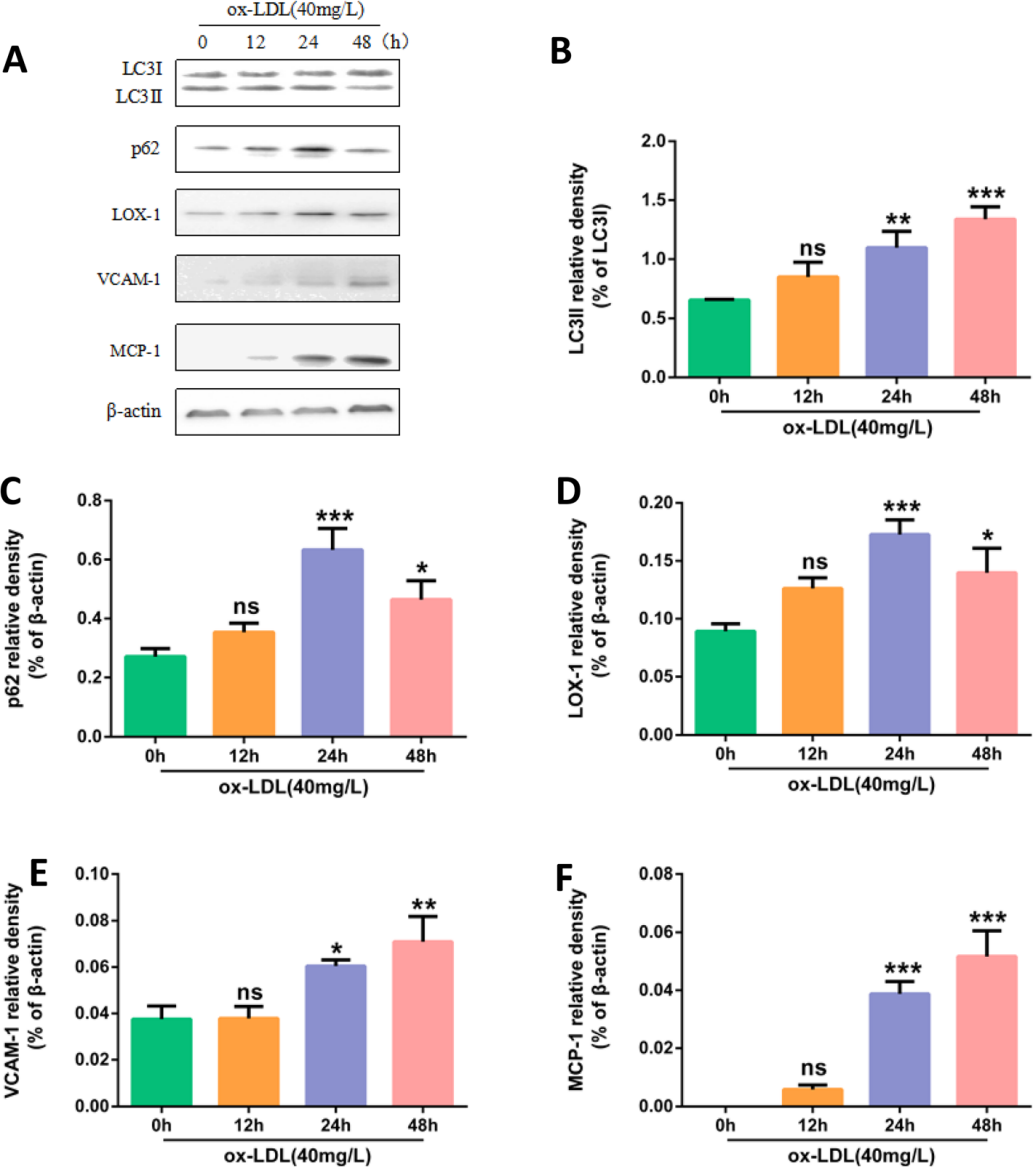
40 mg/L ox-LDL treatment of HUVECs on the expression of inflammation and autophagy proteins at different times. **a** Protein expression of LC3II, p62, LOX-1, VCAM1, MCP-1, β-actin in HUVECs. Cells were treated with 40 mg/L ox-LDL respectively for 0 h,12 h, 24 h, 48 h. **b**, **c**, **d**, **e**, **f** Bar charts show the mean intensity of every protein quantified and normalized versus β-actin expression. Values are submitted as mean ± S.D. based on seperate experiments in triplicate.(*) *P* < 0.05, (**) *P* < 0.01 and (***) *P* < 0.001 versus 0 h

HUVECs were treated with 40 mg/L ox-LDL for 0 h, 12 h, 24 h and 48 h, the results of Western Blot showed that LC3II protein increased in a time-dependent manner, and p62 increased most significantly at 24 h (*P* < 0.001), so in this experiment ox-LDL action time was selected as 24 h. (Fig. 5).

### Ox-LDL induces inflammatory injury of HUVECs

HUVECs were treated with medium containing different concentrations of ox-LDL (0 mg/L, 20 mg/L, 40 mg/L, 60 mg/L, 80 mg/L) for 24 h. The results of Western Blot showed: the expressions of LOX-1, VCAM-1, MCP-1 were all increased. (Fig. 4).

After treated with 40 mg/L ox-LDL for 0 h, 12 h, 24 h and 48 h, Western Blot showed that expressions of VCAM1 and MCP-1 protein increased with the time increase, and LOX-1 expression was the most obvious at 24 h (*P* < 0.001). In this experiment, the ox-LDL action time was selected as 24 h. (Fig. 5).

### Effect of FA on the proliferation of HUVECs

**Fig. 6:**
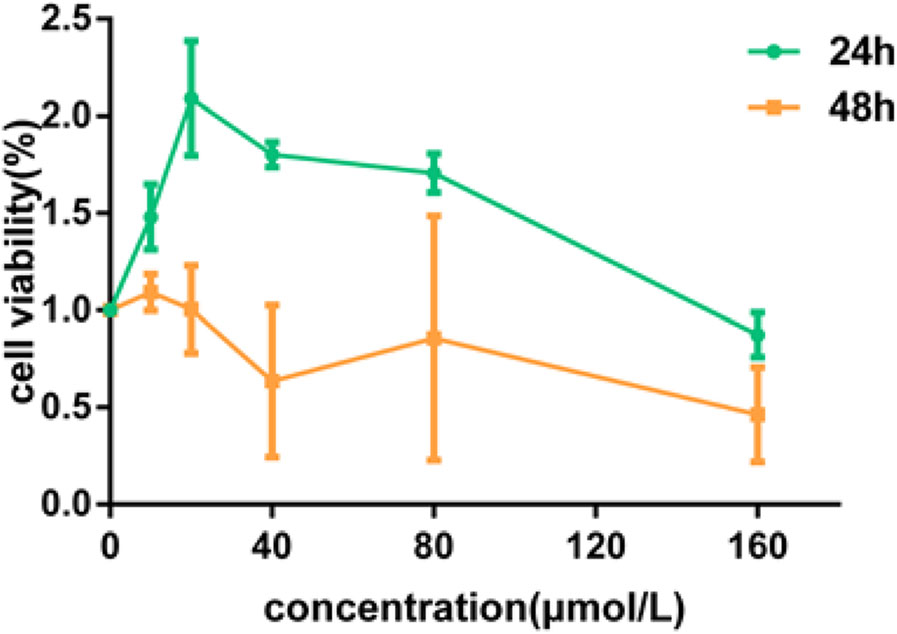
Cell viability was tested by a CCK-8 assay; cell survival was given as percentage of control. HUVECs were respectively treated with FA 10 μmol/L, 20 μmol/L, 40 μmol/L, 80 μmol/L, 160 μmol/L for 24 h and 48 h respectively.

HUVECs were treated with varying concentrations of FA (10 μmol/L, 20 μmol/L, 40 μmol/L, 80 μmol/L, 160 μmol/L) for 24 h and 48 h, respectively. The cell viability was detected by CCK-8 method. The results showed that HUVECs were treated with 20 μmol/L, 40 μmol/L and 80 μmol/L FA for 24 h, and the cell viability was higher than that of 10 μmol/L and 160 μmol/L. There was no significant difference in cell viability among the groups when HUVECs were treated with different concentrations of FA for 48 h (Fig. 6). Therefore, in the subsequent studies, we selected 20 μmol/L, 40 μmol/L, and 80 μmol/L of FA to interfere with HUVECs for 24 h.

### FA increases the level of autophagic flux in HUVECs

**Fig. 7:**
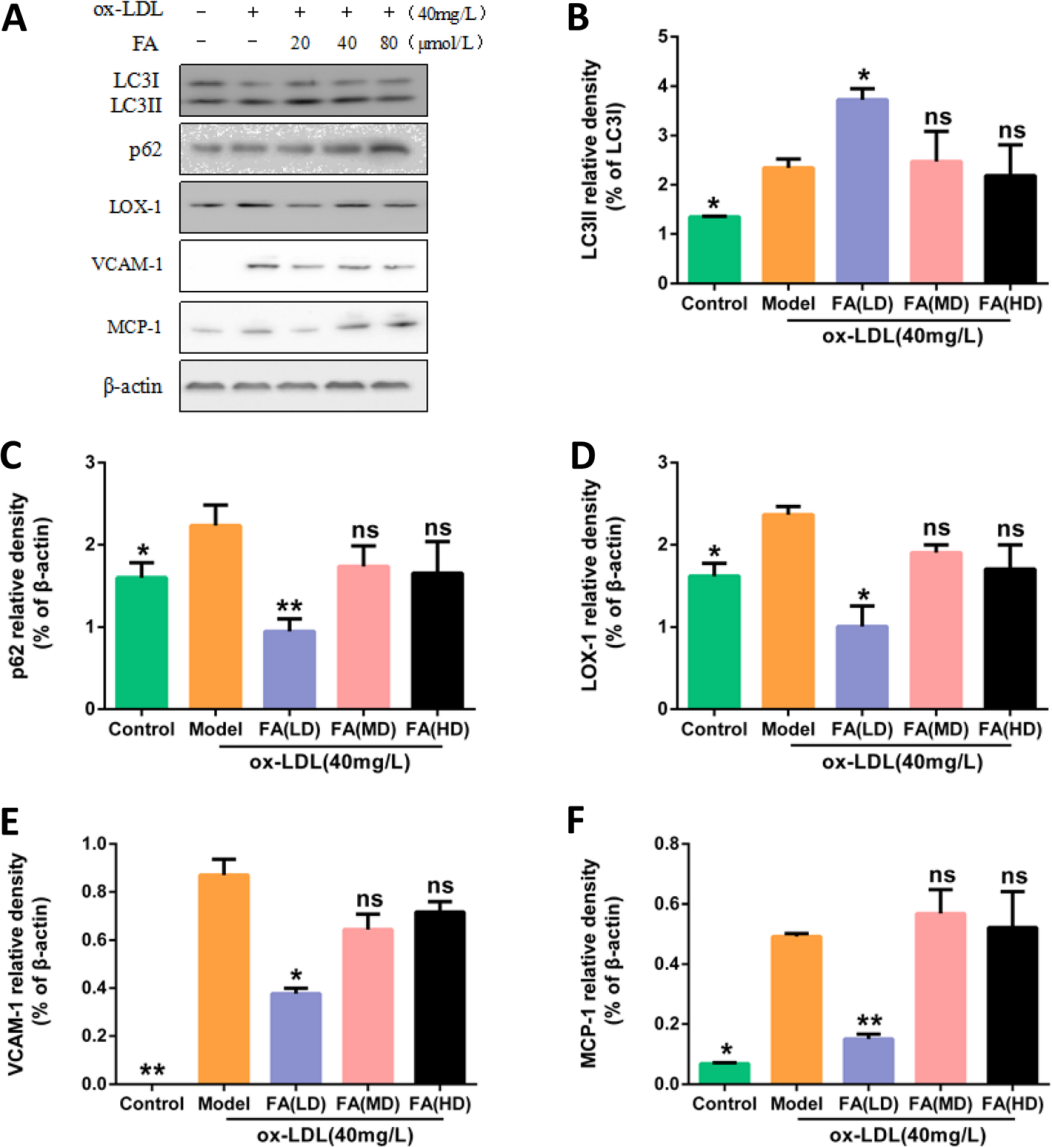
Effect of different concentrations of FA on inflammation and autophagy protein expression. **a** Protein expressions of LC3II, p62, LOX-1, VCAM1, MCP-1, β-actin in HUVECs. The cells were treated with 40 mg/L ox-LDL (model) or not (Control) for 24 h. In addition, cells were pretreated with low dose (LD) 、middle dose (MD) or high dose (HD) of FA for 2 h followed by 40 mg/L ox-LDL exposure for another 24 h. **b**, **c**, **d**, **e**, **f** Bar charts show the mean intensity of every protein quantified and normalized versus β-actin expression. Values are submitted as mean ± SD based on separate experiments in triplicate.(*) *P* < 0.05, (**) *P* < 0.01 versus model

Blank group (complete medium), the ox-LDL model group (40 mg/L ox-LDL), the FA low dose group (20 μmol/L FA pretreatment 2 h + 40 mg/L ox-LDL), FA middle dose group (40 μmol/L FA pretreatment 2 h + 40 mg/L ox-LDL), high dose group FA (80 μmol/L FA pretreatment 2 h + 40 mg/L ox-LDL) groups of cultivating HUVECs 24 h. The expressions of autophagy-related proteins LC3II and autophagy-degrading related proteins p62 were determined by Western Blot. Compared with the blank group, the protein expression of LC3II and p62 increased in ox-LDL model group (*P* < 0.05). Compared with ox-LDL model group, LC3II protein expression increased in FA low dose group (*P* < 0.05), and p62 protein expression decreased significantly (*P* < 0.01).(Fig. 7).

### FA reduces inflammation of HUVECs

Blank group (complete medium), ox-LDL model group (40 mg/L ox-LDL), FA low dose group (20 μmol/L FA pretreatment 2 h + 40 mg/L ox-LDL), FA middle dose group (40 μmol/L FA pretreatment 2 h + 40 mg/L ox-LDL), FA high dose group (80 μmol/L FA pretreatment 2 h + 40 mg/L ox-LDL) groups of cultivating HUVECs 24 h, and the protein expressions of LOX-1, VCAM1, MCP − 1 were detected by Western blot. Compared with the blank group, the protein expressions of LOX-1, VCAM1, MCP-1 was increased in the ox-LDL model group (*P* < 0.05); compared with the ox-LDL model group, the expressions of LOX-1, VCAM1, MCP-1 was significantly reduced in low dose group of FA (*P* < 0.05). (Fig. 7).

### OA increases the level of autophagic flux in HUVECs

**Fig. 8:**
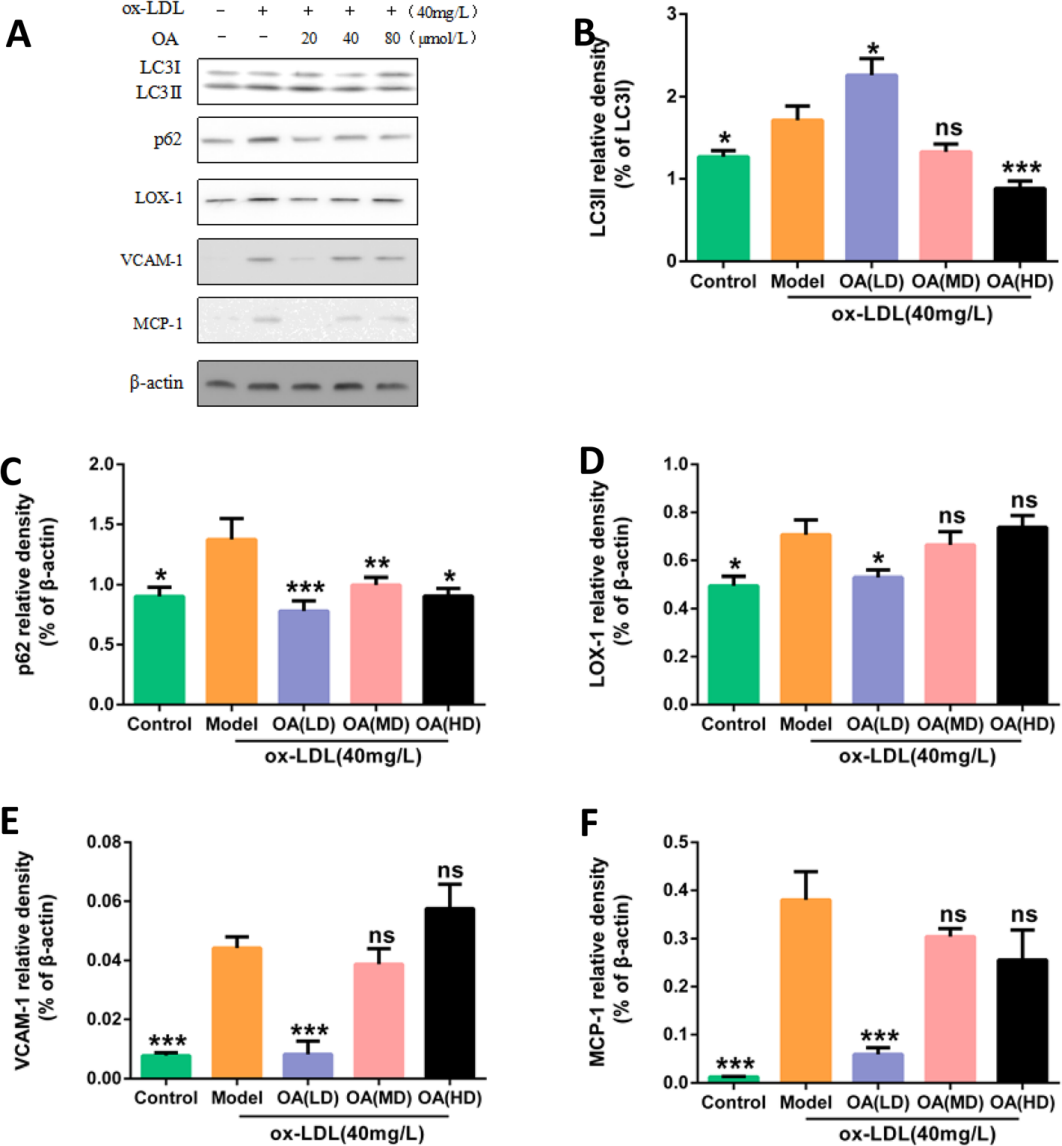
Effects of different concentrations OA on the expression of inflammation and autophagy proteins. **a** Protein expressions of LC3II, p62, LOX-1, VCAM1, MCP-1, β-actin in HUVECs. The cells were treated with 40 mg/L ox-LDL (model) or not (Control) for 24 h. In addition, cells were pretreated with low (LD)、middle dose (MD) or high dose (HD) of OA for 2 h, followed by 40 mg/L ox-LDL exposure for another 24 h. **b**, **c**, **d**, **e**, **f** Bar charts show the mean intensity of each protein quantified and normalized versus β-actin expression. Values are submitted as mean ± SD based on seperate experiments in triplicate.(*) *P* < 0.05, (**) *P* < 0.01 and (***) *P* < 0.001 versus model

Blank group (complete medium), ox-LDL model group (40 mg/L ox-LDL), OA low dose group (20 μmol/L OA pretreatment 2 h + 40 mg/L ox-LDL), OA middle dose group (40 μmol/L OA pretreatment 2 h + 40 mg/L ox-LDL), OA high dose group (80 μmol/L OA pretreatment 2 h + 40 mg/L ox-LDL) cultured HUVECs 24 h, Western Blot was used to test the expression of autophagy-related protein LC3II and autophagy-degrading related protein p62. Compared with the blank group, the expression of LC3II and p62 protein in the ox-LDL model group was increased (*P* < 0.05). Compared with the ox-LDL model group, the expression of LC3II protein in the low dose group of OA was increased (*P* < 0.05), p62 protein significantly decreased expression (*P* < 0.001). (Fig. 8).

**Fig. 9:**
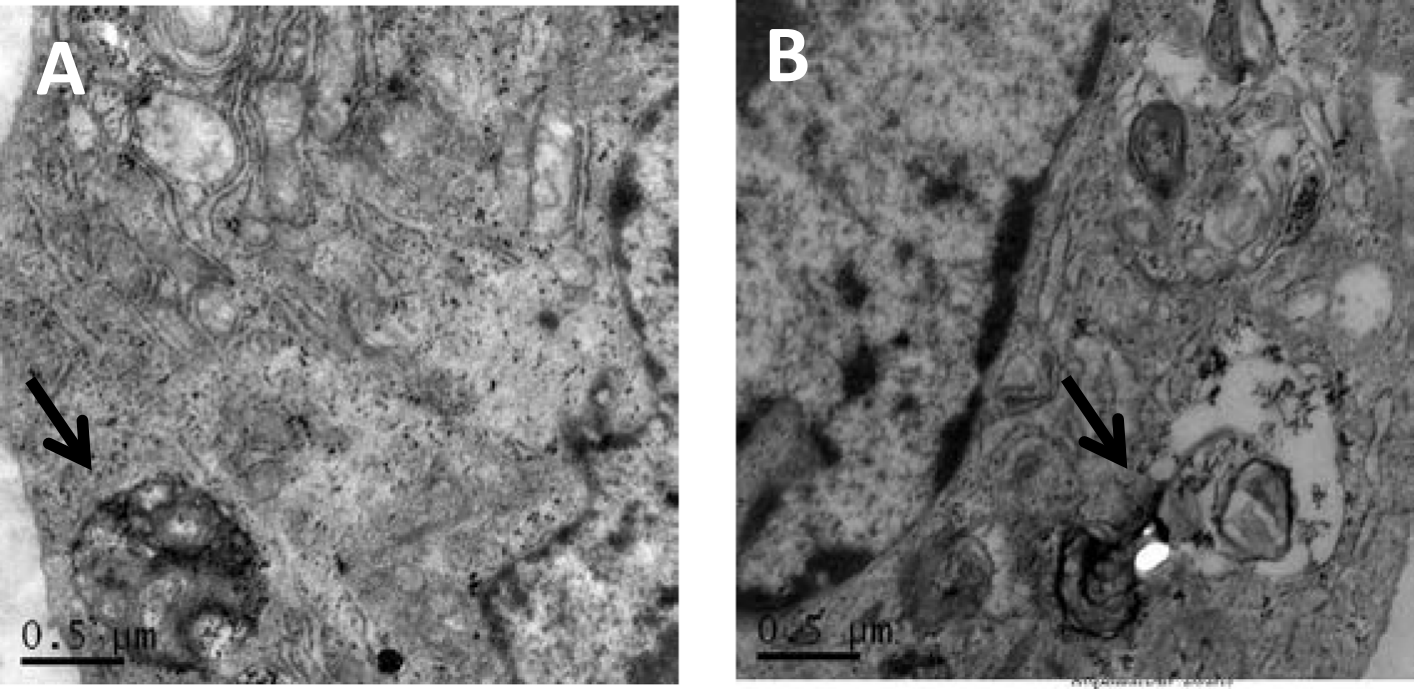
**a** ox-LDL model group (40 mg/L ox-LDL). **b** OA low-dose group, the organelles in the autolysosomes began to degrade, and the produced macromolecules were released through the permeation of cell membranes. Back to the cytoplasm. The black arrow shows autolysosome in the figure. 0.50 μm for all images

HUVECs were observed under TEM. Compared with the ox-LDL model group (40 mg/L ox-LDL), the cells begins to degrade from the autolysosome, and the resulting macromolecule is released back into the cytoplasm through the osmotic action of the cell membrane in the OA low dose group (20 μmol/L OA pretreatment 2 h + 40 mg/L ox-LDL) (Fig. 9).

### OA reduces inflammation of HUVECs

Blank group (complete medium), ox-LDL model group (40 mg/L ox-LDL), OA low dose group (20 μmol/L OA pretreatment 2 h + 40 mg/L ox-LDL), OA middle dose group (40 μmol/L OA pretreatment 2 h + 40 mg/L ox-LDL), OA high dose group (80 μmol/L OA pretreatment 2 h + 40 mg/L ox-LDL) cultured HUVECs 24 h. Western blot was used to detect LOX-1, VCAM1, MCP-1 protein expression. Compared with the blank group, the protein expression of LOX-1, VCAM1, MCP-1 in the ox-LDL model group was increased (*P* < 0.05); compared with the ox-LDL model group, the expressions of LOX-1, VCAM1, MCP- 1 protein was significantly reduced in low dose group of OA (*P* < 0.05). (Fig. 8).

### Effects of FA and OA on autophagy flux of HUVECs

**Fig. 10:**
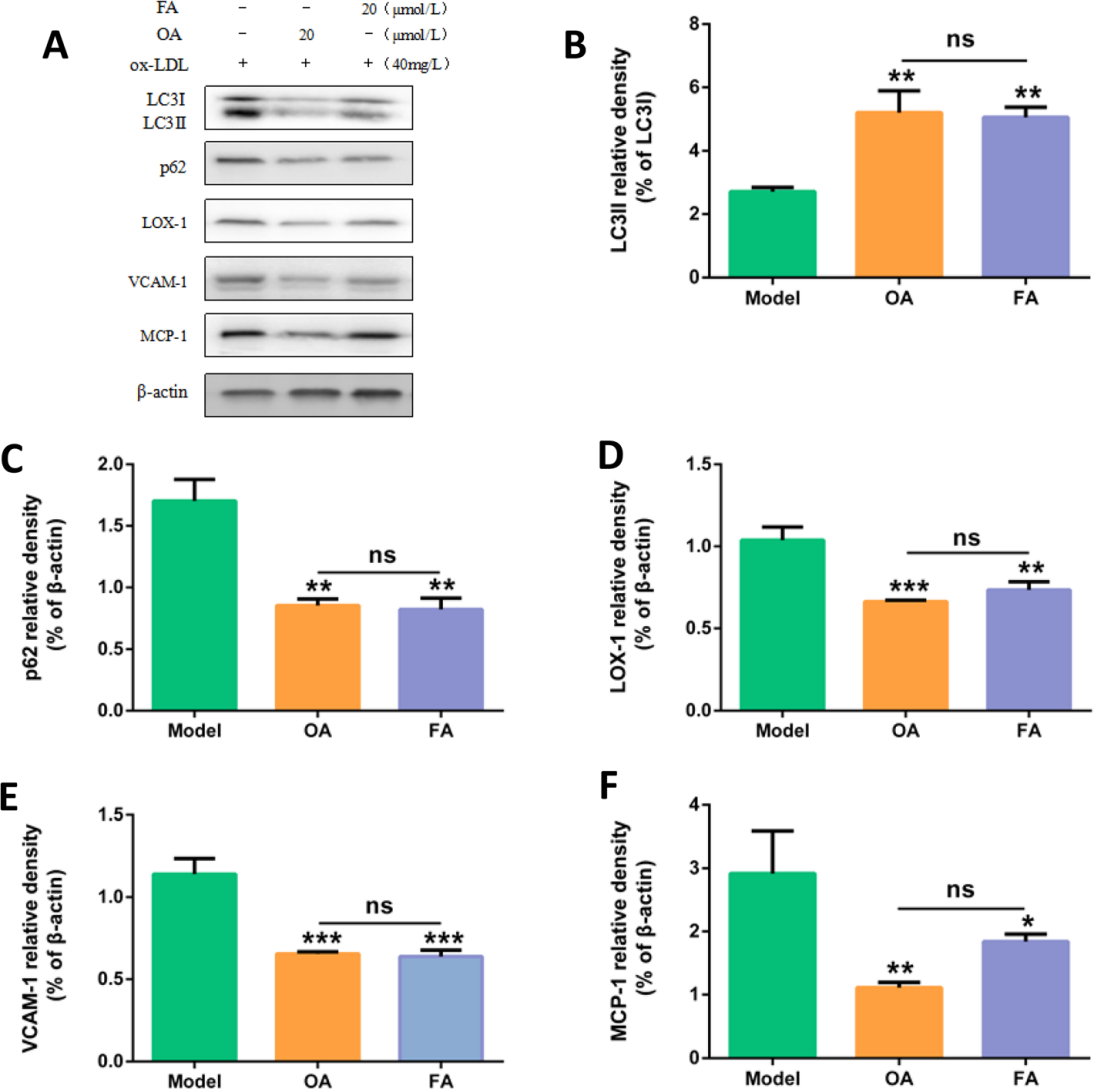
Comparison of protective effects of FA and OA on injured HUVECs. **a** Protein expressions of LC3II, p62, LOX-1, VCAM1, MCP-1, β-actin in HUVECs. The cells were treated with 40 mg/L ox-LDL (model). In addition, cells were pretreated with OA or FA for 2 h followed by 40 mg/L ox-LDL exposure for another 24 h. **b**, **c**, **d**, **e**, **f** Bar charts show the mean intensity of every protein quantified and normalized versus β-actin expression. Values are submitted as mean ± SD based on seperate experiments in triplicate.(*) *P* < 0.05, (**) *P* < 0.01 and (***) *P* < 0.001 versus model

ox-LDL model group (40 mg/L ox-LDL), OA group (20 μmol/L OA pretreatment 2 h + 40 mg/L ox-LDL), FA group (20 μmol/L FA pretreatment 2 h + 40 mg/L ox-LDL). HUVECs were cultured in each group for 24 h, and the expression of autophagy-related protein LC3II and autophagy-degrading protein p62 was detected by Western Blot. Compared with the ox-LDL model group, the expression of LC3II protein in OA and FA groups was increased (*P* < 0.01), and the expression of p62 protein was significantly decreased (*P* < 0.01). There was no significant difference between FA group and OA group (*P* > 0.05). (Fig. 10).

### Effects of FA and OA on inflammation of HUVECs

ox-LDL model group (40 mg/L ox-LDL), OA group (20 μmol/L OA pretreatment 2 h + 40 mg/L ox-LDL), FA group (20 μmol/L FA pretreatment 2 h + 40 mg/L ox-LDL). HUVECs were cultured in each group for 24 h, and the protein expressions of LOX-1, VCAM1, MCP-1 was detected by Western Blot. Compared with the ox-LDL model group, the protein expressions of LOX-1 (*P* < 0.01), VCAM1 (*P* < 0.001), and MCP-1 (*P* < 0.05) in OA group and FA group were significantly decreased. There was no significant difference between FA group and OA group (*P* > 0.05). (Fig. 10).

## Discussion

Atherosclerosis is a serious universal health problem. ox-LDL induced vascular endothelial cells injury is the driving force for its initiation and progression [11, 12]. The vascular lumen stenosis and hemorheology abnormality caused by the formation of atherosclerotic plaque are similar to the “blood stasis syndrome” in Chinese medicine [13]. Therefore, drugs for treating blood stasis may play an important role in the early stage of atherosclerosis development. It has shown that Chinese materia medica for promoting blood circulation and removing blood stasis may exert anti-atherosclerotic effects through anti-inflammatory, anti-oxidation, lipid-lowering and endothelial protection. Among the organic acids of Angelica sinensis, the high content of FA is the main molecule of pharmacological action at this site. FA is a natural phenolic acid with anti-inflammatory, anti-oxidative, anti-platelet aggregation effects, and has been used in experimental studies against atherosclerosis.

Autophagy is a self-phagocytic pathway that promotes cell survival in adverse environments. The researches showed that autophagy is involved in the progression of atherosclerosis [14]. However, the stimulation of autophagy is only beneficial if the autophagy flux is not compromised [15]. In this study, we induced endothelial injury model by ox-LDL and found that ox-LDL can cause autophagic flux damage in HUVECs and cause endothelial cell inflammatory response. We pretreated HUVECs with angelica organic acid and found that angelica organic acid can enhance the autophagy flux and reduce the inflammatory response.

Autophagy is a fundamental metabolism process in which impaired or aging organelles can be removed to maintain a basic homeostasis [16, 17]. In atherosclerosis, in vitro studies indicate that autophagy is present in all related cell types, just as macrophages, vascular endothelial cells, and vascular smooth muscle cells. Successful autophagy can promote cells survival and reverse cells damage and dysfunction [18, 19]. Autophagy is even considered to be a negative regulator of inflammatory responses and oxidative stress. Autophagy may also improve the stability of atherosclerotic plaque in the body, slow the development of plaque, and prevent plaque rupture [20]. Based on these findings, autophagy is helpful in stabilizing atherosclerotic vulnerable plaque.

Normally, autophagy is fulfilled through the autophagosome formation, which includes the conversion of cytosolic LC3-I to LC3-phosphatidylethanolamine conjugate (LC3-II). Therefore the LC3-II/LC3-I ratio is often used as a quantitative indicator for autophagy [21, 22]. The current main way for monitoring autophagy activation is to detect LC3 by Western blot and to detect the formation of autophagosomes by electron microscopy. In the process of ox-LDL-induced endothelial cells inflammatory damage, we found that with the increase of ox-LDL concentration and time, the level of LC3II protein associated with autophagy increased and the number of autophagosomes increased significantly. However, autophagy is a greatly dynamic, multi-step process. Amassing of autophagosomes may indicate occlusion of autophagy activation or downstream steps of autophagy, such as reduced fusion of autophagosomes with lysosomes or reduced lysosomal degradation [23]. Therefore, only detecting the number of autophagosomes and LC3 levels does not allow a comprehensive and accurate assessment of the entire autophagy system, as increased numbers of autophagosomes and up-regulation of LC3II protein may be due to excessive autophagy activation or reduced autophagy degradation [24].

In the later stages of autophagy, LC3II may be degraded by SQSTM1/p62, which means the integrity of the autophagy process. P62 is involved in ubiquitination and proteasome degradation in the nucleus. Accumulation of p62 reflects a decrease in autophagic degradation, while low levels of p62 indicate activation of autophagy degradation [25–27]. In the process of ox-LDL-induced endothelial cells inflammatory damage, we found that expression of p62 increased when 40 mg/L ox-LDL was applied to HUVECs. Transmission electron microscopy showed that when 40 mg/L ox-LDL was applied to HUVECs, the autophagosomes increased significantly, and the damaged organelles in autophagosomes showed no degradation. This indicates that ox-LDL can induce autophagy activation, but when 40 mg/L ox-LDL is applied to HUVECs, the efficiency of autolysosome degradation is reduced, and the number of damaged organelles accumulated in the cells is increased. This may lead to or worsen atherosclerosis.

In order to study how OA play an anti-atherosclerosis role by regulating autophagy in HUVECs. We pretreated HUVECs with OA and FA for 2 h, and then cultured HUVECs with 40 mg/L ox-LDL for 24 h, and then to test the changes of autophagy-related proteins LC3II/LC3I, p62 and the structure of autophagosomes. We found that the LC3II protein expression was up-regulated and the p62 protein expression was down-regulated in the 20 μmol/L OA and FA groups compared with the model group. It indicated that both low dose OA and FA could activate autophagy of endothelial cells and increase the level of autophagy degradation, thereby increasing the autophagy flux and accelerating the degradation of damaged organelles in HUVECs, which may be beneficial to the stability of plaque and delay the atherosclerosis process.

The development of atherosclerotic plaque can be attributed to endothelial dysfunction caused by long-term inflammatory responses. Therefore, how to protect the endothelial function of artery is crucial for the pathogenesis of atherosclerosis. In the site of vascular endothelial injury, the transport of ox-LDL is considered to be an important starting event for atherosclerosis [28]. LOX-1 is the ox-LDL receptor in endothelial cell, a combination of ox-LDL and LOX-1 induced endothelial activation and dysfunction [29], local inflammation of blood vessels, induce endothelial cells expressed VCAM 1, make the mononuclear cells and T lymphocyte adhesion in the damaged endothelial surface, MCP-1 will receive the induction signal to get them into the lining, to recruit mononuclear cells to the area of inflammation, and through clearance of the endothelial lining quickly migrated to the subendothelial area. This process triggers the foam cells formation, promotes the smooth muscle cells migration and proliferation, and leads to the formation of fibrous plaques. With the inflammatory cells infiltration and the lipids deposition, the fibrous cap gradually became thinner and eventually evolved into unstable plaques [30–32]. In this experiment, the expressions of VCAM-1, MCP-1 and LOX-1 in endothelial cells were up-regulated by ox-LDL, which proved that ox-LDL induced endothelial cells inflammatory damage. We pretreated HUVECswith OA and FA for 2 h, then cultured HUVECs with 40 mg/L ox-LDL for 24 h, and detected inflammation-related proteins VCAM-1, MCP-1 and LOX-1. We found that the levels of VCAM-1, MCP-1, and LOX-1 proteins were significantly down-regulated in the 20 μmol/L OA group and the FA group compared with the model group. It is indicated that both low dose of OA and FA have anti-inflammatory and protective effects on endothelial cells.

Finally, in order to compare the anti-atherosclerosis effect of OA and FA, we pretreated HUVECs with 20 μmol/L OA and FA for 2 h, then cultured HUVECs with 40 mg/L ox-LDL for 24 h to detect autophagy-related proteins and inflammation-related proteins expression. We found that, compared with the model group, both OA and FA not only increased the activation of autophagy in endothelial cells, but also increased the level of autophagy degradation. Thereby, they can increase the autophagy flux. They had the effect of reducing inflammation and protecting endothelial cells. However, there was no statistical difference between the OA group and the FA group (*P* > 0.05). Therefore, we speculated that the main anti-AS component of OA was FA.

Based on the above results, we found that OA may exert anti- atherosclerosis effects by enhancing the autophagic flux of the cells and alleviating the inflammatory response. But the relationship between the two aspects remains to be further studied. Regarding the relationship between autophagy and inflammation, it has been suggested that autophagy protects cells from long-lasting inflammatory effects by at least two methods: (1) indirectly by removing damaged organelles (such as mitochondria) or intracellular pathogenic microorganisms, (2) directly through the inhibition of inflammatory bodies [33, 34]. There is also a correlation between autophagy and inflammatory body: ①autophagy has a negative regulatory effect on the activation of inflammatory body; ②autophagy induction depends on the existence of certain inflammatory body sensors;③the inflammatory body is finally degraded by autophagosome through selective autophagy receptor p62 [35, 36].

As we know, three compounds besides ferulic acid have been isolated from the water extract of Radix Angelica sinensis, and their structures were elucidated as Z-6,7-cis-dihydroxyligustilide, bis (2-ethylhexyl) phthalate and brefeldin A3 [37]. These compounds are very minor and not enough to investigate their contribution to the activity of the water extract of Angelica. In the near future work, we will isolate and accumulate these minor compounds or synthesize these compounds for accurately analyzing their effects on autophagy dysfunction of HUVECs as well as their synergistic action to completely characterize the potential benefit of this extract.

## Conclusion

In this experiment, we selected HUVECs as the tool cells, and found that OA can increase the autophagic flux of endothelial cells by detecting autophagy-specific proteins LC3, p62 and autophagosome. OA also can down-regulate the inflammation-related proteins VCAM-1 and MCP-1, LOX-1, therefore, we speculate that OA plays a role in the prevention and treatment of atherosclerosis by enhancing the flux of autophagic cells and reducing the inflammatory response. However, its specific mechanism of action needs further research.

## Data Availability

The datasets generated and/or analysed during the current study are available from the corresponding author on reasonable request.

## Abbreviations

OA: Organic acid
HPLC: High-performance liquid chromatography
FA: Ferulic acid
HUVECs: Human umbilical vein endothelial cells
Ox-LDL: Oxidized low-density lipoprotein
TEM: Transmission electron microscope
LDL: Low density lipoprotein
WB: Western blot
AS: Atherosclerosis
NF-κB: Nuclear factor kappa-light-chain-enhancer of activated B cells
ROS: Reactive oxygen species
VCAM-1: Vascular cell adhesion molecule-1
MCP-1: Monocyte chemotactic protein-1
LC3: Microtubule-associated protein 1 light chain 3
P62: Sequestosome 1
Bcl-2: b-cell lymphoma-2
CCK-8: Cell Counting Kit
